# Lightweight Cross-Domain Few-Shot Plant Disease Recognition Through Target-Domain Statistical Calibration

**DOI:** 10.3390/s26123632

**Published:** 2026-06-07

**Authors:** Chuantao Zhao, Ting Xu, Zhixian Zhang, Xia Geng

**Affiliations:** College of Information Science and Engineering, Shandong Agricultural University, Tai’an 271018, China; chuantaozhao1@outlook.com (C.Z.); 19550877687@163.com (T.X.); hanxin6667@gmail.com (Z.Z.)

**Keywords:** cross-domain few-shot learning, plant disease recognition, target-domain BN adaptation, prototypical learning, lightweight edge deployment

## Abstract

**Highlights:**

**What are the main findings?**

- Under a unified PlantVillage (PV_100)-to-PlantDoc split protocol, the proposed training-time target-domain BN adaptation (TBA) strategy achieved cross-split mean accuracies of 42.69 ± 0.62% for one-shot and 54.24 ± 0.72% for five-shot, where ± denotes the standard deviation across three independent data splits.
- The main performance gains stemmed from TBA; adding DANN or TSA-style test-time adaptation did not yield consistent additional improvements.

**What are the implications of the main findings?**

- For agricultural cross-domain few-shot disease recognition, shifting the main domain-correction step to training is more reliable than adding complex adaptation procedures at test-time.
- The encoder with the default no-mask pooling branch achieved full NPU execution, with an estimated single-sample inference latency as low as 658 μs, highlighting its potential for encoder-level mobile deployment.

**Abstract:**

Plant disease recognition models trained under laboratory conditions often degrade markedly after cross-domain transfer because of the pronounced distribution gap between source and target domains and the scarcity of labeled target-domain samples. To address the transfer task from PlantVillage (PV_100) to PlantDoc, this study develops and evaluates a lightweight cross-domain few-shot plant disease recognition method under a strict PlantVillage-to-PlantDoc protocol. The method integrates EfficientNet-B0 feature extraction, cosine-similarity-based prototypical classification, and training-time target-domain BN adaptation (TBA). During training, unlabeled target-domain images are used only for BN statistical calibration, whereas inference is limited to feature extraction and prototype matching, without gradient updates or iterative optimization. Under a unified experimental protocol, the proposed method achieved cross-split mean accuracies of 42.69 ± 0.62% for one-shot and 54.24 ± 0.72% for five-shot, where ± denotes the standard deviation across three strict data splits; it outperformed ProtoNet by 7.44 and 9.43 percentage points, respectively. Ablation results indicate that TBA is the main source of performance improvement, whereas more complex adaptation strategies do not yield stable additional gains. The core encoder can be executed entirely on the NPU, with an estimated single-sample inference latency as low as 0.658 ms, indicating strong potential for encoder-level mobile deployment.

## 1. Introduction

Plant disease recognition is essential for early disease warning and crop management decisions, and deep learning methods have achieved encouraging results under controlled conditions [1,2,3,4]. In real field scenarios, however, images are often affected by complex backgrounds, illumination changes, occlusion, and camera-to-object distance variations; consequently, models transferred from laboratory settings to field environments tend to suffer substantial performance degradation [1,2,3]. Agricultural applications also face a persistent shortage of labeled data, especially for long-tail diseases and newly emerging regional scenarios, where sufficient annotations cannot be collected within a short period. Recent studies have therefore explored few-shot plant disease recognition under limited-label settings [5,6,7,8,9], while lightweight and edge-device-based models have also been investigated for practical agricultural diagnosis [10,11]. Few-shot learning (FSL) provides an important means of alleviating annotation scarcity, yet its effectiveness often depends on the assumption that the training and test domains follow similar distributions, which is difficult to satisfy in agricultural cross-domain settings.

Taking the transfer from PlantVillage [12] to PlantDoc [13] as an example, the pronounced distribution gap between laboratory images and real field images can weaken the discriminative ability of the learned feature space [2]. In the few-shot learning literature, methods such as ProtoNet, Matching Networks, Relation Networks, and MAML have shown that metric learning or meta-learning can support recognition of novel classes under extremely limited supervision [14,15,16,17]. Methods such as DeepBDC further attempt to enhance discriminative ability by using more complex statistical representations [18]. However, most of these methods have been validated either in same-domain settings or under relatively weak domain shift, and their stability in agricultural cross-domain scenarios remains limited.

To narrow the discrepancy between the source and target domains, prior studies have introduced feature transformation, self-training, adversarial augmentation, adaptive transformer-based adaptation, feature-space adaptation, loss-landscape flattening, general feature learning, and open-set domain-adaptation methods for plant disease recognition [19,20,21,22,23,24,25,26]. During testing, methods such as TENT, TTN, MemBN, DELTA, NanoAdapt, and Fourier-based adaptation attempt to further reduce distribution shift through online statistic updates, entropy minimization, or frequency-domain compensation [27,28,29,30,31,32,33]. These training-time and test-time adaptation methods often incur higher computational and storage costs, making them less suitable for mobile edge deployment. Plant disease recognition commonly relies on fine-grained cues such as leaf texture, lesion boundaries, and local color variation; overly aggressive feature alignment or test-time optimization may disturb such discriminative information and therefore produce unstable gains or even negative transfer.

Motivated by these considerations, this study adopts a lightweight strategy in which low-level statistics are calibrated using unlabeled target-domain images during training, whereas testing is restricted to feature extraction and prototype matching, without gradient updates or iterative optimization. Built upon EfficientNet-B0 [34] and prototypical classification, the proposed method uses training-time target-domain BN adaptation (TBA) to update target-domain Batch Normalization (BN) statistics [35], thereby correcting differences in imaging style, background, and illumination at relatively low cost. Because TBA adjusts only normalization statistics rather than actively reshaping the feature-space structure, it is better suited to preserving the fine-grained cues required for plant disease recognition. Meanwhile, domain-adversarial alignment [36] and TSA-style test-time spectral adaptation are included as additional analyses to examine whether more complex adaptation strategies can outperform TBA when agricultural cross-domain few-shot learning and mobile deployment constraints coexist. The focus of this work is to compare the practical gains and costs of different adaptation strategies under the same experimental conditions.

Although EfficientNet-B0, prototypical classification, and BN-based statistical adaptation are established components, each component alone addresses only part of the PlantVillage-to-PlantDoc challenge. EfficientNet-B0 provides a compact encoder but does not by itself correct laboratory-to-field distribution shift; prototypical classification supports few-shot novel-class recognition but remains sensitive to domain-shifted embeddings; and BN statistical calibration can reduce low-level imaging mismatch but does not alone constitute a complete cross-domain few-shot recognition method. Therefore, this study develops a lightweight cross-domain few-shot recognition method that combines compact feature extraction, prototype-based target-class recognition, and training-time target-domain statistical calibration. Under a strict protocol in which unlabeled target images are kept disjoint from final test samples and test-time optimization is avoided, the proposed method provides a low-cost solution for agricultural field diagnosis under scarce target labels and deployment constraints.

The main contributions of this study are as follows:

(1) A strict and reproducible data-splitting and evaluation protocol is established for the cross-domain scenario between PlantVillage and PlantDoc. The protocol explicitly distinguishes source-domain training classes, source-domain validation classes, an unlabeled target-domain image pool, and a target-domain novel-class test set. Training-time unlabeled target-domain images are kept strictly separate from the final test samples, and healthy overlapping classes are excluded from the novel-class test set by default, thereby enabling a more reliable evaluation of disease generalization.

(2) A lightweight cross-domain few-shot recognition method is developed for the joint constraints of scarce target labels, laboratory-to-field domain shift, and test-time efficiency. The method combines EfficientNet-B0 feature extraction, cosine-similarity-based prototype classification, and training-time target-domain BN statistical calibration, so that target-domain imaging statistics are calibrated before inference while the test stage remains limited to feature extraction and prototype matching.

(3) Extensive experiments, including reproduced baselines, ablation studies, DANN and TSA-style controls, cross-split stability analysis, error analysis, and encoder-level mobile profiling, show that the proposed method provides more stable gains than source-only training, representation-level adversarial alignment, or test-time spectral adaptation under the strict PlantVillage-to-PlantDoc protocol. The deployment-oriented analysis further demonstrates encoder-level feasibility for lightweight edge deployment.

## 2. Materials and Methods

### 2.1. Datasets

The source domain is PlantVillage [12], and the target domain is PlantDoc [13]. PlantVillage mainly provides relatively clean images captured under controlled conditions, whereas PlantDoc more closely reflects real field acquisition and involves complex backgrounds, scale variation, and illumination perturbation. Based on these two datasets, this study formulates a cross-domain few-shot recognition task from PlantVillage to PlantDoc, and the filtered, split-specific PlantVillage source subset is denoted as PlantVillage (PV_100). Let the source domain consist of labeled base-class data, and let the target domain contain two parts: an unlabeled image pool for TBA and a target-domain novel-class sample pool for episode-level evaluation.

Under this task formulation, the base classes in PlantVillage are used for episodic learning during training, whereas the non-healthy disease classes in PlantDoc that are semantically shared with PlantVillage form the novel-class test set. Under the default setting, overlapping healthy classes are excluded from the novel-class test set.

The PlantVillage-to-PlantDoc setting was selected because it provides a representative and reproducible laboratory-to-field transfer scenario for plant disease recognition, which is consistent with recent studies on plant disease recognition in the wild and cross-domain few-shot crop disease identification [1,2]. PlantVillage is a public plant disease dataset covering multiple crop species and disease categories, with images mainly captured under relatively controlled conditions and clean backgrounds [12]. In contrast, PlantDoc contains field-oriented plant disease images covering multiple crops and disease categories, where leaves often appear with complex backgrounds, illumination variation, scale changes, partial visibility, and non-standard acquisition conditions [13]. Because the two datasets share semantically related disease categories but differ strongly in acquisition conditions, their combination provides a suitable source-to-target setting for evaluating laboratory-to-field cross-domain few-shot recognition. This source-to-target construction reflects a common deployment pathway in practical agricultural diagnosis, where models trained from curated or controlled image resources must be adapted to less controlled mobile or field images collected by farmers, extension workers, or field phenotyping platforms. Therefore, this protocol captures a core and reproducible form of agricultural domain shift: transfer from curated laboratory-style disease images to less controlled field images, while maintaining a controlled evaluation setting for comparing different methods.

**Figure 1:**
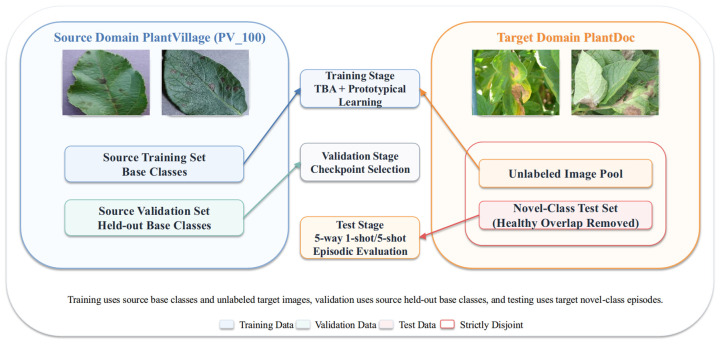
Schematic illustration of the data split and evaluation workflow between PlantVillage (PV_100) and PlantDoc.

The data split and evaluation workflow are shown in Figure 1.

### 2.2. Method

**Figure 2:**
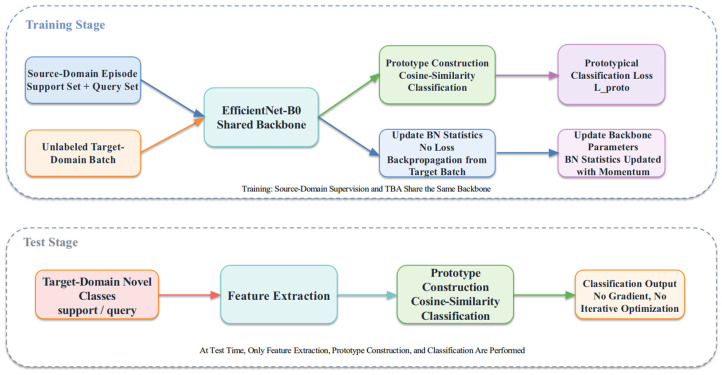
Overall framework of the proposed method.

As shown in Figure 2, the proposed method uses EfficientNet-B0 as the backbone and performs prototypical learning on source-domain episodes. During training, unlabeled target-domain images are used to update BN statistics so as to alleviate the impact of cross-domain imaging differences. During testing, only prototype-based classification is performed, without gradient updates or iterative optimization.

#### 2.2.1. Lightweight Backbone and Prototype Classification

EfficientNet-B0 is the smallest model in the EfficientNet family and was designed using compound scaling, which jointly balances network depth, width, and input resolution [34]. Its main building blocks are mobile inverted bottleneck convolutions combined with squeeze-and-excitation modules, providing a favorable trade-off between representation capacity and computational cost. Because of this efficiency, EfficientNet-B0 has been widely adopted as a lightweight feature extractor in visual recognition tasks, including medical image analysis, remote sensing, industrial inspection, and agricultural disease recognition. In this study, it is used as the backbone to keep the encoder compact while retaining sufficient visual representation ability for fine-grained plant disease cues.

For an input image x, the backbone network $f_{\theta }\left(\cdot \right)$ (EfficientNet-B0 [34]) outputs a feature map $F\in R^{C\times H\times W}$. In the setting adopted in this work, global average pooling is used to obtain an embedding vector $z\in R^{C}$:

(1) $$z=GAP\left(F\right)$$

For simplicity, the backbone and pooling operation are jointly denoted as the embedding extractor $g_{\theta }\left(x\right)$.

Within each episode, class prototypes are constructed from support embeddings following the ProtoNet formulation [14]:

(2) $$p_{c}=\frac{1}{\left|S_{c}\right|}\sum_{(x,y)\in S_{c}}g_{\theta }\left(x\right)$$

where $S_{c}$ is the support subset for class c. The support embeddings, query embeddings, and prototypes are all $L_{2}$-normalized to improve cross-domain robustness. The normalized vectors are denoted by $\hat{z}$ and $\hat{p}$. For a query sample $x_{q}$, logits are computed using cosine similarity with a temperature coefficient τ, which is fixed at τ=10.0 in this study:

(3) $$l_{q,c}=\tau \cdot {\hat{z}}_{q^{⊤}}{\hat{p}}_{c}$$

The final episodic classification loss is the cross-entropy over the query set:

(4) $$L_{proto}=E_{\left(x_{q},y_{q}\right)\in Q}\left[CE\left(l_{q},y_{q}\right)\right]$$

The combination of a lightweight backbone and prototypical classification offers three advantages. First, prototype construction relies directly on mean statistics of the support set, making the method simple and interpretable. Second, cosine similarity together with normalized embeddings helps alleviate the impact of inconsistent feature scales across domains. Third, the test stage requires no additional optimization of the classification head, which makes the approach well suited to resource-constrained scenarios. In this work, it serves as the base recognizer, allowing the practical gains introduced by domain-adaptation modules to be evaluated more clearly.

#### 2.2.2. Training-Time Target-Domain BN Adaptation (TBA)

In the current task, the source and target domains exhibit substantial statistical shifts in imaging style, illumination, and background. The goal is therefore to calibrate the target domain at minimal additional cost while minimizing disturbance to the original feature-space structure, so as to preserve the fine-grained cues required for disease recognition. On this basis, TBA is introduced in addition to prototypical classification. During training, unlabeled target-domain images that are strictly disjoint from the final test samples are passed through the network to update the running mean and variance of BN layers, enabling the discriminative parameters learned on the source domain to operate more reliably under target-domain imaging conditions. The entire procedure focuses on statistical calibration and does not introduce complex semantic reconstruction of the target domain.

For an arbitrary BN layer, let the running statistics formed during source-domain supervised training be $\left(\mu_{s},\sigma_{s^{2}}\right)$, and let the statistics estimated from an unlabeled target-domain batch be $\left(\mu_{t},\sigma_{t^{2}}\right)$. For an input activation x, the affine normalization of standard BN can be written as

(5) $$\hat{x}=\gamma \frac{x-\mu }{\sqrt{\sigma^{2}+\varepsilon }}+\beta$$

where γ and β are learnable affine parameters of the layer, and ε is a numerical-stability term. In the proposed TBA procedure, γ and β are kept as the parameters learned by source-domain episodic supervised training, while the unlabeled target-domain data are used only to update the running statistics. The BN transformation after TBA is therefore written as

(6) $${\hat{x}}_{t}=\gamma \frac{x-\mu_{t}}{\sqrt{\sigma_{t^{2}}+\varepsilon }}+\beta$$

This formulation means that the model retains the channel-scaling and bias information learned under source-domain supervision while replacing the normalization reference with target-domain statistics. Because γ and β remain unchanged, the relative scaling relationships among feature channels are preserved, which helps maintain the fine-grained discriminative patterns learned from the source domain. Meanwhile, the normalization reference is adapted to the target-domain distribution, thereby mitigating feature shifts caused by imaging-style differences.

In implementation, the unlabeled target-domain batch is used only to update BN running statistics and is not involved in loss computation or backpropagation. The update follows the original momentum setting of the BN layers in the backbone network. For EfficientNet-B0, the BN momentum is 0.1, allowing target-domain statistics to accumulate gradually from the unlabeled data stream without being dominated by a single small batch. The additional overhead of TBA is incurred mainly during training and does not introduce iterative optimization at test-time, making the method more compatible with on-device deployment constraints.

### 2.3. Training Strategy

For the proposed method, the training objective consists solely of the source-domain episodic prototypical classification loss:

(7) $$L=L_{proto}$$

where $L_{proto}$ is always computed from the query samples in the source-domain episode to maintain class discrimination, whereas the unlabeled target-domain batch is used only to update BN statistics and does not participate in loss backpropagation. During training, only $D_{s}$ and $D_{t^{u}}$ are used, and no labeled PlantDoc novel-class samples are involved; those labeled samples appear only as support or query samples in the final test episodes. Training therefore involves two data streams: source-domain episodic supervision and unlabeled target-domain batches used for TBA. The model first samples an episode from the source-domain base classes and computes $L_{proto}$, and then samples a batch from the unlabeled target-domain image pool for an additional forward pass that updates BN running statistics.

Training uses the AdamW optimizer with a budget of 30 epochs × 200 steps per epoch. Input images are resized to 224 × 224, converted to RGB tensors, and normalized using ImageNet statistics.

At test-time, model parameters are kept fixed and no gradient updates are performed. For each episode, class prototypes are first constructed from the support samples, after which the query samples are classified to compute accuracy. For each single run, episode-level results are reported over 1000 episodes as the mean accuracy and 95% confidence interval. The entire test process includes only EfficientNet-B0 feature extraction and prototype matching, without iterative optimization.

### 2.4. Experimental Environment and Settings

To ensure a fair comparison, the main result tables include only methods evaluated under a unified training strategy, data split, and evaluation pipeline, rather than directly juxtaposing published results obtained under inconsistent protocols or without a unified validation procedure. The main methods compared in the paper include ProtoNet, CDFSL-NML [2], CDFSL-MAML [2], CDFSL-BDC [2], and the proposed method. These methods represent several common few-shot recognition paradigms, including prototype-based metric learning, nearest-mean classification, gradient-based meta-learning, and second-order statistical representation learning. Results for the Matching-, Relation-, and FWT-style variants are reported in Appendix A to complement the performance of other paradigms under the same experimental conditions; the corresponding key settings and additional results are listed in Table A1 and Table A2.

The main experimental environment and settings are listed in Table 1 and Table 2.

Except for algorithm-specific modules, all remaining implementation details are kept as consistent as possible to reduce the influence of engineering factors on the conclusions. All methods are trained and evaluated on three strict data splits, and cross-split statistics are reported. During training, validation is performed once at the end of each epoch, and the best checkpoint is selected using validation episodes constructed from the held-out base classes of PlantVillage. The validation stage uses 100 episodes, and the final test stage uniformly uses 1000 episodes. The PlantDoc novel classes used for formal testing do not participate in training supervision or model selection.

### 2.5. Evaluation Metrics

Episode-level classification accuracy is used as the primary evaluation metric. For a single training/evaluation run, the average accuracy and the 95% confidence interval (mean ± 95% CI) are computed over 1000 episodes, where the 95% confidence interval is computed as $1.96\times std\left(acc\right)/\sqrt{N}$ with N=1000.

For the main result tables and the multi-split ablation analysis, cross-split mean ± standard deviation across the three strict data splits is reported to reflect the combined variation caused by split differences and training randomness. Thus, mean ± 95% CI denotes within-run uncertainty over episodes, whereas mean ± standard deviation denotes cross-split reproducibility; these two statistics are not interchangeable.

## 3. Results and Analysis

### 3.1. Comparison with Unified Baselines

Table 3 summarizes the results obtained under three strict splits, a unified training budget, and a unified evaluation pipeline. To facilitate overall comparison, only the cross-split mean ± standard deviation is reported.

As shown in Table 3, the proposed method achieves 42.69 ± 0.62% for one-shot and 54.24 ± 0.72% for five-shot, which are the best results in the table. Compared with ProtoNet, the proposed method improves performance by 7.44 and 9.43 percentage points in the one-shot and five-shot settings, respectively. Relative to NML, BDC, and MAML, the gains range from 6.94 to 14.07 percentage points for one-shot and from 8.26 to 22.41 percentage points for five-shot. Under identical experimental conditions, TBA yields stable performance gains.

### 3.2. Cross-Split Stability Analysis

**Figure 3:**
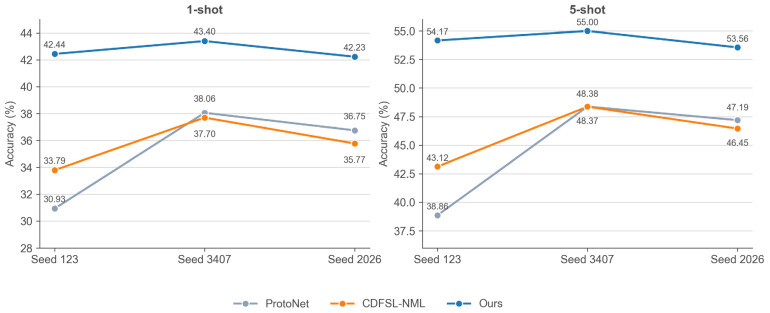
Accuracy variation of ProtoNet, CDFSL-NML, and the proposed method across three independent data splits.

ProtoNet, CDFSL-NML, and the proposed method exhibit different degrees of performance fluctuation across the three independent splits, as shown in Figure 3.

Figure 3 shows that the proposed method achieves one-shot accuracies of 42.44%, 43.40%, and 42.23% across the three splits, and five-shot accuracies of 54.17%, 55.00%, and 53.56%, with standard deviations of only 0.62 for one-shot and 0.72 for five-shot. By contrast, ProtoNet shows standard deviations of 3.80 and 5.19, whereas CDFSL-NML shows standard deviations of 1.96 and 2.66. The proposed method not only achieves a higher mean accuracy but also yields more consistent performance across different data splits. TBA reduces the model’s dependence on a specific class split and thereby improves robustness.

### 3.3. Ablation Study

Table 4 presents the ablation results for the key components.

Table 4 shows that, compared with the prototype baseline without TBA, introducing TBA increases the average accuracy across the three strict splits from 35.25%/44.81% to 42.69%/54.24%, corresponding to gains of 7.44 and 9.43 percentage points for one-shot and five-shot, respectively. This result indicates that TBA is the primary source of improvement under the current cross-domain setting.

This study also compares the mask and no-mask pooling branches. Enabling the mask branch yields cross-split averages of 40.84 ± 3.41% for one-shot and 51.95 ± 4.30% for five-shot, both lower than those of the default no-mask configuration and accompanied by larger fluctuations, suggesting that this branch does not provide consistent benefits.

Table 4 also reports the DANN control results. Compared with the TBA-only configuration, adding DANN yields average results of 42.12%/53.02%, without further improvement and with larger cross-split variation.

**Figure 4:**
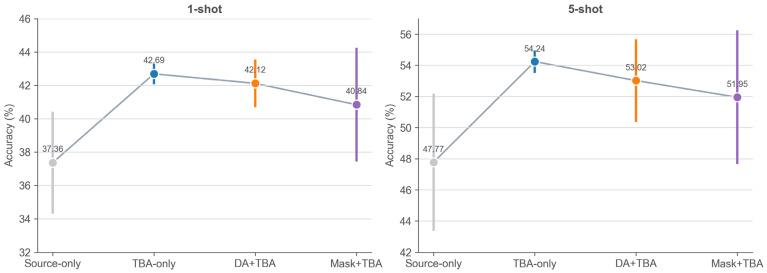
Ablation comparison among the source-only, TBA-only, and DA+TBA configurations.

The ablation trends shown in Table 4 are consistent with those in Figure 4.

**Figure 5:**
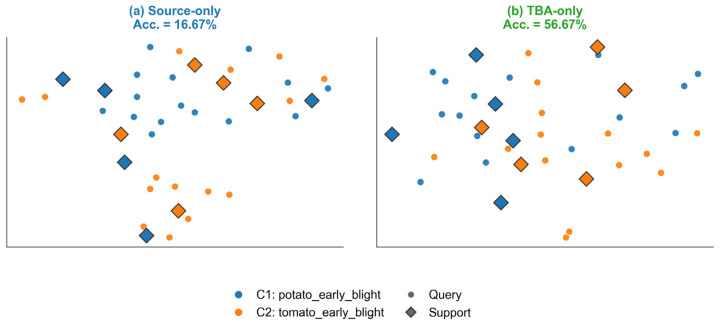
Qualitative t-SNE visualization of source-only and TBA-only on a fixed binary episode from the target domain.

To further visualize the influence of TBA on the feature distribution, Figure 5 provides a qualitative t-SNE visualization using a fixed target-domain binary episode. This binary setting is used only for visualization clarity and is separate from the formal five-way evaluation protocol.

As shown in Figure 5, when the two novel classes ‘potato_early_blight’ and ‘tomato_early_blight’ are used as a binary visualization example, the target-domain feature points are heavily mixed under the source-only setting, whereas TBA-only yields much clearer inter-class separation. This qualitative observation is consistent with the ablation results in Table 4.

### 3.4. TSA-Style Test-Time Spectral Adaptation

Table 5 reports the results of the TSA-style test-time spectral adaptation strategy. These results are based on evaluation over 1000 episodes using a single seed (123). Because the statistical protocol differs from the three-split averaging used in Table 3 and Table 4, this section is intended mainly to illustrate relative trends.

Table 5 indicates that, for the proposed method, adding TSA changes the one-shot accuracy from 42.44% to 42.28% and the five-shot accuracy from 54.17% to 54.33%. For BDC, the changes are similarly marginal, from 35.19% to 35.32% for one-shot and from 43.94% to 43.98% for five-shot. Overall, TSA does not deliver stable accuracy improvements. Compared with these negligible accuracy changes, the additional time overhead introduced by TSA is more apparent. For the proposed method, the test-time per episode increases from 2.11 s to 2.13 s, whereas for BDC it increases from 2.11 s to 2.14 s. When the gain is close to zero, such additional overhead has limited practical value.

**Figure 6:**
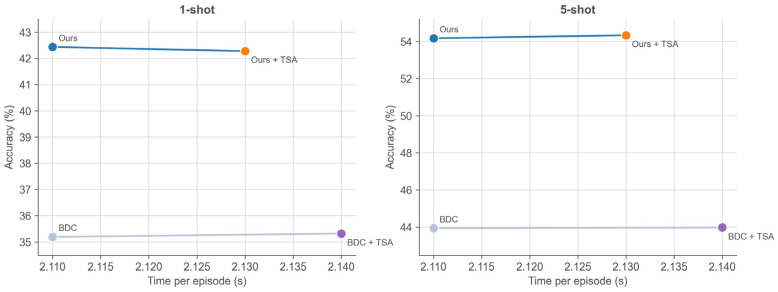
Accuracy–efficiency trade-off of TSA-style test-time spectral adaptation.

The accuracy–efficiency relationship shown in Figure 6 further reflects this trend.

These results indicate that, in the present task, more complex test-time adaptation is not necessarily superior to lightweight training-time adaptation. For plant disease recognition scenarios that emphasize stability and controllable latency, additional test-time processing is worthwhile only when it provides a clear accuracy benefit.

### 3.5. Error Analysis and Practical Reliability

Although the proposed method improves the average accuracy over the reproduced baselines, the absolute accuracies of 42.69% for one-shot and 54.24% for five-shot indicate that the PlantVillage-to-PlantDoc task remains highly challenging. To better understand the remaining failure modes, an error analysis was conducted over the three strict splits. For each shot setting, the analysis included 225,000 query predictions in total, obtained from 1000 episodes per split, five classes per episode, and 15 query images per class. For each query image, the ground-truth class and predicted class were recorded to compute class-wise accuracy and episodic confusion statistics.

Table 6 shows substantial variation among disease categories. The two corn disease classes achieved the highest accuracies, and ‘grape_black_rot’ also showed relatively strong performance, especially in the five-shot setting. These categories tend to contain more distinctive color or texture patterns, making prototype-based discrimination more reliable. By contrast, ‘tomato_leaf_mold’, ‘apple_scab’, ‘tomato_late_blight’, ‘potato_early_blight’, ‘potato_late_blight’, and ‘tomato_bacterial_spot’ remained difficult. The limited improvement from one-shot to five-shot for some categories, especially ‘tomato_leaf_mold’, suggests that the remaining errors are not caused only by the number of support samples, but also by lesion similarity, intra-class variation, background clutter, and residual domain shift.

**Figure 7:**
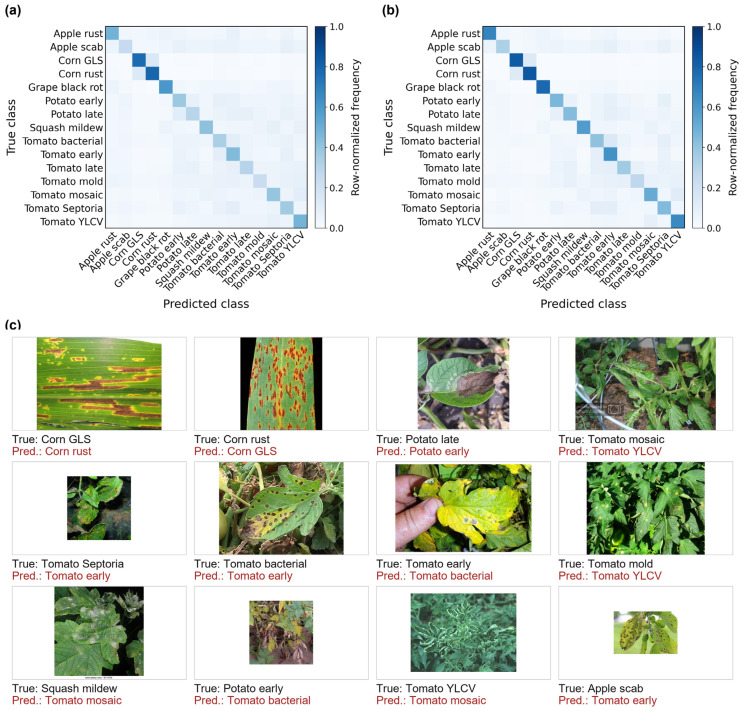
Error analysis of the proposed method. (**a**) Episodic confusion matrix under the 1-shot setting. (**b**) Episodic confusion matrix under the 5-shot setting. (**c**) Representative misclassified PlantDoc images.

The confusion matrices in Figure 7a,b further support this interpretation. These matrices are computed under episodic five-way evaluation; therefore, each query prediction is made among the five classes sampled in the corresponding episode rather than among all target classes simultaneously. The most frequent one-shot confusions included ‘corn_cercospora_leaf_spot_gray_leaf_spot’ being predicted as ‘corn_common_rust’, ‘corn_common_rust’ being predicted as ‘corn_cercospora_leaf_spot_gray_leaf_spot’, ‘tomato_septoria_leaf_spot’ being predicted as ‘tomato_early_blight’, ‘tomato_mosaic_virus’ being predicted as ‘tomato_yellow_leaf_curl_virus’, and ‘potato_late_blight’ being predicted as ‘potato_early_blight’. Similar patterns remained in the five-shot setting, especially among tomato diseases and between the two corn disease categories. These errors indicate that the model often fails when different diseases share similar local lesion morphology or when field backgrounds and scale variation weaken the representativeness of support prototypes.

The representative misclassified images in Figure 7c show complex field backgrounds, partial leaves, small lesion regions, uneven illumination, and symptoms occupying only a limited portion of the image. These observations suggest that the proposed method is most suitable for lightweight screening and decision support, while more robust class-wise discrimination is still needed for standalone field diagnosis.

### 3.6. Mobile Deployment Analysis

**Figure 8:**
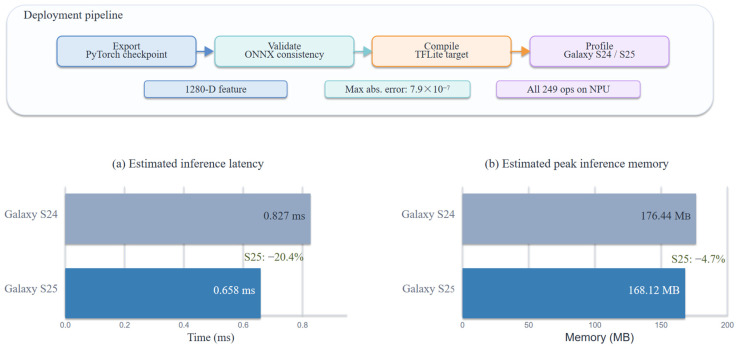
Deployment pipeline and mobile performance analysis of the encoder with the default no-mask pooling branch.

The deployment encoder corresponding to the proposed method, namely the EfficientNet-B0 backbone together with global pooling and normalized output, was exported to ONNX (Linux Foundation, San Francisco, CA, USA) and further compiled into TFLite. Cloud-based real-device profiling was then carried out on Samsung Galaxy S24 and Samsung Galaxy S25 devices (Samsung Electronics Co., Ltd., Suwon, Republic of Korea). The relevant results are shown in Table 7 and Figure 8. The maximum absolute difference between the ONNX and PyTorch outputs is only 7.9 × 10^−7^, indicating reliable numerical consistency after export.

Table 7 shows that both the initial estimated latency and the steady-state average latency reported by AI Hub are low on both devices. All 249 operators are mapped to the NPU on both devices. The estimated single-sample inference latency is approximately 0.827 ms on S24 and 0.658 ms on S25, while the corresponding steady-state average latency is 0.851 ms and 0.676 ms, respectively. Peak inference memory is 176.44 MB on S24 and 168.12 MB on S25.

The first-load time is approximately 0.90 s on S24 and 0.59 s on S25, and the warm-load time is approximately 0.17 s and 0.10 s, respectively. These results indicate that the encoder can achieve sub-millisecond single-sample inference latency on mainstream mobile SoCs and therefore has promising on-device deployment feasibility.

As shown in Figure 8, the workflow forms a deployment chain from ONNX export and TFLite compilation to mobile performance profiling, while also presenting the latency and memory differences between S24 and S25.

This deployment analysis verifies encoder-level on-device feasibility rather than full on-device deployment of the complete few-shot recognition pipeline. Prototype construction from support samples, support/query scheduling, memory usage during episodic inference, and the accuracy–efficiency trade-off after INT8 quantization remain to be systematically evaluated under a unified hardware and inference backend. The current results demonstrate that the core encoder has strong potential for edge deployment, and future system-level work can extend this profiling to full episodic inference with prototype management, memory analysis, and quantization-aware optimization.

## 4. Discussion

The results have several implications for agricultural cross-domain few-shot recognition and deployment. First, the stable gains of TBA suggest that low-cost statistical calibration can be more suitable than heavier adaptation modules when the target-domain labels are scarce and the test stage must remain simple. In the PlantVillage-to-PlantDoc task, the discrepancy between laboratory images and field images is manifested mainly in low-level statistical factors such as imaging style, illumination, background, and scale perturbation, while disease recognition still depends on fine-grained cues such as leaf texture, lesion boundaries, and local color changes. Because TBA updates only BN statistics rather than actively reshaping the feature-space structure, it can reduce target-domain imaging shift while causing less interference with disease-discriminative patterns learned from the source domain. This also helps explain why DANN and TSA-style test-time adaptation did not provide reliable additional benefits in the present experiments.

The present results suggest that TBA is particularly suitable when the main domain discrepancy comes from low-level imaging statistics and the disease-related texture or lesion patterns remain partially shared between the source and target domains. In this case, adapting target-domain BN statistics can improve the feature distribution without introducing additional test-time optimization. However, statistical calibration alone may be insufficient when the main errors arise from visually similar diseases, very small or occluded lesions, or support samples that do not adequately represent the full intra-class variation of the target categories. These failures suggest that future improvements should focus on stronger local discriminative representation and prototype refinement for visually similar diseases and small-lesion cases.

Second, the PlantVillage-to-PlantDoc protocol provides a reproducible way to study a practical acquisition-to-recognition gap in image-based disease recognition. Models trained mainly from controlled source images must operate on target images collected under less controlled field conditions. This setting is also related to broader image-based high-throughput plant phenotyping pipelines, where image acquisition, field acquisition variability, data processing, model inference, and downstream decision support are closely connected. Recent discussions on image-based plant phenotyping have emphasized field imaging variability, limited annotations, model generalization, and deployable inference as important challenges for scalable agricultural applications [37].

Third, the results support a deployment-oriented adaptation route: unlabeled target-domain images are used to complete statistical calibration during training, while the test stage retains only feature extraction and prototype matching. The encoder-level deployment analysis further supports this point. The core encoder can achieve full NPU execution on Samsung Galaxy S24/S25, with an estimated single-sample inference latency of approximately 0.83 ms and 0.66 ms, respectively. Recent resource-efficient agricultural diagnosis models, such as RF-Cott-Net [38], also highlight the importance of jointly considering accuracy, model size, latency, and deployability in field-oriented recognition. In relation to this trend, the present study focuses on cross-domain few-shot recognition with scarce target labels, strict episodic evaluation, training-time statistical calibration, and encoder-level mobile feasibility. In real field workflows, such predictions could serve as a lightweight front-end for preliminary screening and decision support, together with image-quality control, confidence estimation, expert confirmation, and field management feedback.

This study has two main limitations. First, the current experiments focus mainly on the PlantVillage-to-PlantDoc transfer scenario, and the compared CDFSL-NML, MAML, and BDC methods are reproduced under the same experimental conditions. Although this setting provides a controlled and representative benchmark for analyzing laboratory-to-field transfer, evaluation on additional public datasets and real field collections would further test the robustness of the method under more diverse crop species and acquisition conditions. The proposed method is not inherently restricted to the crops evaluated in this study, but its practical use on new crops requires labeled source-domain disease images, a small number of target-domain support samples, and unlabeled target-domain images for BN statistical calibration.

Second, although TBA yields stable improvements, the absolute accuracy in the one-shot setting remains limited, class-wise performance varies substantially, and the current deployment analysis is confined to encoder-level validation. Future work will focus on two directions: improving class-wise robustness through lesion-aware representation learning and prototype refinement, and extending mobile evaluation from encoder profiling to full episodic inference with memory and quantization analysis. These extensions would further connect lightweight few-shot recognition with real field screening and agricultural decision support.

## 5. Conclusions

For the cross-domain few-shot plant disease recognition task between PlantVillage and PlantDoc, this study proposes a lightweight prototypical learning method centered on TBA. The method is built upon EfficientNet-B0 and prototypical classification, performs TBA during training using unlabeled target-domain images that are strictly disjoint from the final test samples, and relies only on feature extraction and prototype matching during testing.

Under three strict data splits, a unified training budget, and a unified evaluation protocol, the proposed method achieves average results of 42.69 ± 0.62% for one-shot and 54.24 ± 0.72% for five-shot, outperforming the reproduced ProtoNet, CDFSL-NML, CDFSL-MAML, and CDFSL-BDC baselines under the same experimental conditions. Ablation studies further show that the performance improvement mainly comes from TBA, whereas adding DANN or TSA-style test-time adaptation does not yield stable additional gains.

Overall, TBA provides an effective and low-cost technical route for the current agricultural cross-domain few-shot recognition task. It improves cross-domain recognition performance without introducing additional test-time optimization steps, while remaining compatible with encoder-level mobile deployment constraints. Combined with the encoder-level full NPU execution results on Samsung Galaxy S24/S25, the proposed method provides empirical support for the design and deployment of lightweight models in intelligent agricultural diagnosis.

## Figures and Tables

**Table 1:** Main experimental environment.

Item	Setting
Operating system	Windows 10 Home China (25H2) (Microsoft Corporation, Redmond, WA, USA)
Python/PyTorch/CUDA	Python 3.12.3 (Python Software Foundation, Beaverton, OR, USA)/PyTorch 2.10.0 (PyTorch Foundation, San Francisco, CA, USA)/CUDA 12.8 (NVIDIA Corporation, Santa Clara, CA, USA)
GPU	NVIDIA GeForce RTX 4060 Laptop GPU, 8 GB (NVIDIA Corporation, Santa Clara, CA, USA)

**Table 2:** Main experimental settings.

Item	Setting
Backbone network	EfficientNet-B0
Input size	224 × 224
Training task setting	5-way, 5-shot support, 15 queries/class
Test task setting	5-way, 1-shot/5-shot, 15 queries/class
Data split	3 splits (123, 3407, 2026)
Validation scale	100 episodes per epoch
Final test scale	1000 episodes

**Table 3:** Comparison of different methods on the PlantVillage (PV_100)-to-PlantDoc cross-domain task.

Method	1-Shot (%)	5-Shot (%)
ProtoNet [14]	35.25 ± 3.80	44.81 ± 5.19
CDFSL-NML [2]	35.75 ± 1.96	45.98 ± 2.66
CDFSL-MAML [2]	28.62 ± 3.70	31.83 ± 5.80
CDFSL-BDC [2]	33.50 ± 1.71	42.74 ± 1.05
Ours (no-mask, TBA-only)	42.69 ± 0.62	54.24 ± 0.72

Note: Values are reported as the mean ± standard deviation across three strict splits.

**Table 4:** Ablation results of key components across three strict splits.

Variant	1-Shot (%)	5-Shot (%)
Source-only (Proto, no TBA, no DANN)	35.25 ± 3.80	44.81 ± 5.19
TBA-only (Proto + TBA)	42.69 ± 0.62	54.24 ± 0.72
TBA-only (mask pooling)	40.84 ± 3.41	51.95 ± 4.30
DA+TBA (Proto + DANN + TBA)	42.12 ± 1.43	53.02 ± 2.66

Note: Values are reported as the mean ± standard deviation across three strict splits.

**Table 5:** Accuracy–efficiency comparison for TSA-style test-time spectral adaptation.

Method	1-Shot (%)	5-Shot (%)	Test-Time per Episode (s)
Ours (no TSA)	42.44 ± 0.65	54.17 ± 0.64	2.11
Ours + TSA	42.28 ± 0.65	54.33 ± 0.64	2.13
BDC (no TSA)	35.19 ± 0.59	43.94 ± 0.59	2.11
BDC + TSA	35.32 ± 0.61	43.98 ± 0.61	2.14

Note: Results are based on a single-model evaluation with seed = 123 and are reported as mean accuracy ± 95% confidence interval over 1000 episodes. This statistical protocol differs from the mean ± standard deviation across three data splits used in Table 3 and Table 4 and is intended primarily for analyzing the accuracy–efficiency trade-off of test-time adaptation. The time values indicate the end-to-end test-time per episode.

**Table 6:** Representative class-wise accuracies averaged over three strict splits.

Class	1-Shot (%)	5-Shot (%)
corn_cercospora_leaf_spot_gray_leaf_spot	77.61	85.04
corn_common_rust	76.91	84.41
grape_black_rot	55.77	74.98
tomato_leaf_mold	26.06	31.42
apple_scab	26.88	34.35
tomato_late_blight	30.22	37.82
potato_early_blight	31.07	37.36
potato_late_blight	31.61	43.93
tomato_bacterial_spot	32.41	39.44

**Table 7:** Mobile performance analysis of the deployment encoder on Samsung Galaxy S24/S25.

Device	Format	Estimated Inference Time (μs)	Mean Steady Inference Time (μs)	First Load (μs)	Warm Load (μs)	Peak Inference Memory (MB)	Operators on NPU
Samsung Galaxy S24	TFLite	827	851	902,446	170,176	176.44	249/249
Samsung Galaxy S25	TFLite	658	676	585,527	101,734	168.12	249/249

Note: The latency values in the table follow the original AI Hub unit of ‘μs’, and are converted to ‘ms’ in the main text. Estimated inference time denotes the initial latency estimate, whereas mean steady inference time denotes the average steady-state latency.

## Data Availability

PlantVillage and PlantDoc are publicly available datasets. The unified PlantVillage (PV_100)-to-PlantDoc split files, training and evaluation scripts, and model weights used in this study are available from the corresponding author on reasonable request.

## Appendix A. Additional Results and Supporting Information

This appendix provides additional variant results and relevant setting descriptions under the unified training framework. These materials are intended to illustrate the behavior of different paradigms under the shared protocol and are not used as the primary comparative evidence in the main text.

**Table A1:** Key settings of the methods included in the main result table.

Method	Training Paradigm	Key Settings (Excluding the Backbone)
ProtoNet (reproduced under our setting)	Episodic prototypical learning	No domain-adversarial alignment or TBA; no-mask pooling
CDFSL-NML (reproduced under our setting)	Source-domain supervised pretraining + episodic prototype inference	Supervised pretraining batch size = 64; all other evaluation protocols follow those of the proposed method
CDFSL-MAML (reproduced under our setting)	Episodic meta-learning	Default inner-loop setting; model selection is uniformly based on the source-domain validation set
CDFSL-BDC (reproduced under our setting)	Episodic metric learning	BDC embedding dimension = 128
Ours (no-mask, TBA-only)	Episodic prototypical learning + TBA	No domain-adversarial alignment; unlabeled target-domain batches are used only to update BN statistics; no-mask pooling

Note: This table lists only the key items affecting fairness of comparison and is intended to clarify the training paradigm and core settings of each method.

**Table A2:** Results of additional variants at seed = 123.

Method (Additional Variant, Seed = 123)	1-Shot (%)	5-Shot (%)
Matching-style template	33.12 ± 0.52	42.40 ± 0.55
Relation-style template	33.84 ± 0.56	42.83 ± 0.53
FWT-style template	39.75 ± 0.61	50.34 ± 0.63

Note: This table supplements the performance of several template-based variants under the unified evaluation protocol and is not used as a core comparison table in the main text.
